# In Vivo Non-Invasive High-Resolution Imaging for the Evaluation of the Periocular Skin Area: A Comprehensive Review of the Literature

**DOI:** 10.3390/jcm15041571

**Published:** 2026-02-17

**Authors:** Camilla Chello, Giuseppe Paolo Antonio Gemma, Riccardo Sadun, Luca Ambrosio, Elisabetta Angela Campanale, Simone Cappilli, Giovanni Pellacani

**Affiliations:** Dermatology Clinic, Department of Medical and Cardiovascular Sciences, Sapienza University of Rome, 00185 Rome, Italy; camilla.chello@gmail.com (C.C.); giuseppe.gemma@uniroma1.it (G.P.A.G.); sadun.riccardo@gmail.com (R.S.); luca.ambrosio@uniroma1.it (L.A.); elisabettaangela.campanale@uniroma1.it (E.A.C.); pellacani.giovanni@gmail.com (G.P.)

**Keywords:** digital imaging, skin cancers, diagnosis, clinical dermatology, optical coherence tomography, reflectance confocal microscopy, LC-OCT

## Abstract

The periocular region represents a highly sensitive anatomical area due to its functional relevance and aesthetic importance. It is frequently affected by a broad spectrum of cutaneous tumors, due to chronic exposure to ultraviolet radiation, hence an accurate diagnosis and lesion margin assessment is essential to guide appropriate treatment. Herein we summarize the current evidence on the use of reflectance confocal microscopy (RCM), optical coherence tomography (OCT), and line-field confocal optical coherence tomography (LC-OCT) for the assessment of periocular skin tumors. A comprehensive literature search was conducted in the main databases following PRISMA 2020 guidelines. Studies published between 2015 and 2025 evaluating the application of RCM, OCT, and LC-OCT in skin tumors of this area were included. RCM was the most extensively studied modality, demonstrating utility in the characterization of pigmented and non-pigmented periocular lesions and in the identification of basal cell carcinoma-specific features. OCT provided complementary information by enabling visualization of deeper tissue structures, particularly in non-melanoma skin cancers; LC-OCT showed high concordance with histopathology providing practical advantages in this area. As a conclusion, non-invasive imaging techniques represent valuable tools in the evaluation of periocular skin tumors, as they may enhance diagnostic confidence and support clinical decision-making.

## 1. Introduction

A wide spectrum of conditions may involve the periocular region, from benign to malignant tumors and inflammatory and infectious diseases [1,2]. Given the high functional and cosmetic relevance of this anatomical region, even small lesions may result in disproportionate morbidity, making early and accurate diagnosis imperative. Indeed, the complex anatomy of the periocular unit features primarily a thin skin, dense vascularization, and the proximity to vital structures like the eye, lacrimal system, and orbital septum. Although benign cutaneous lesions are the most frequently encountered in this area, the differential diagnosis with malignant counterparts often represents a clinical challenge [2,3,4]. Subtle clinical presentations and unclear dermoscopic patterns may potentially lead to delayed recognition of early malignant lesions. Moreover, the limited possibility of performing wide surgical margins excisions in this district underscores the need for highly reliable preoperative diagnostic tools. This issue is particularly relevant as the periocular area is a highly sensitive anatomical region that deserves special consideration for the maintenance of the structural and aesthetic integrity [3,4]. Among malignant skin tumors herein occurring, basal cell carcinoma (BCC) is the most commonly reported, followed by squamous cell carcinoma (SCC), cutaneous melanoma (CM), and sebaceous gland carcinoma (SGC) [2,4].

In addition to these entities, the local presence of several adnexal and periocular structures may give rise to less common, site-specific malignancies that increase diagnostic complexity [4]. In routine clinical practice, diagnosis is commonly based on clinical and dermoscopic features, even though additional non-invasive techniques, where available, should be applied in equivocal cases, as these tools may provide complementary morphological information, thereby improving diagnostic accuracy and clinician confidence [5,6,7,8]. In this scenario, in vivo non-invasive imaging modalities have progressively acquired a role in the conventional examination, as these technologies align with the paradigm that shift toward a precision diagnostic process, where accuracy must be maximized while reducing unnecessary biopsies and surgical procedures. This aspect is of particular importance if we consider that treatment of malignancies in this area is of interdisciplinary relevance, not so often managed by different specialist figures involving oculofacial surgeons, radiation oncologists, medical oncologists and dermatologists [9]. An accurate pre-treatment assessment has a mainstay importance for an integrated therapeutic planning, since a delay in diagnosis or a misdiagnosis may affect the function of the eye, with a meaningful impact on the patient’s vision and quality of life [2,4,8,9]. While surgery remains the gold standard, in particular Mohs micrographic surgery and other forms of Peripheral and Deep En Face Margin Assessment (PDEMA), recent advances in systemic neoadjuvant therapies (immunotherapy and targeted therapy) are revolutionizing treatment by potentially sparing patients from disfiguring procedures like orbital exenteration.

In this review we summarize the literature evidence focusing on the application of the most used in vivo non-invasive imaging techniques for the diagnosis of skin tumors in the periocular area. They are mainly reflectance confocal microscopy (RCM), optical coherence tomography (OCT), and the new line-field confocal optical coherence tomography (LC-OCT). LC-OCT is a recently marketed in vivo device characterized by advanced technical properties that have recently attracted increasing scientific interest [8,10]. It can acquire high-definition images in vertical and horizontal modes, merging RCM cellular resolution (~1 μm) and OCT depth acquisition (~500 μm) [8,10], thus offering the potential for a comprehensive, real-time “virtual biopsy” of the examined area. By combining high cellular resolution with deeper penetration, LC-OCT represents a promising step toward the potential reduction of the need for invasive diagnostic procedures. However, up to date, the role of LC-OCT has limited validation.

The integration of these imaging techniques into routine clinical workflows may enhance early detection, improve surgical planning, and promote tissue-sparing approaches, thereby preserving both visual function and aesthetic outcomes.

Despite the growing availability of these technologies, their specific role and comparative performance in the periocular setting remain insufficiently standardized and scattered across the literature.

## 2. Materials and Methods

### 2.1. Search Strategy

**Figure 1:**
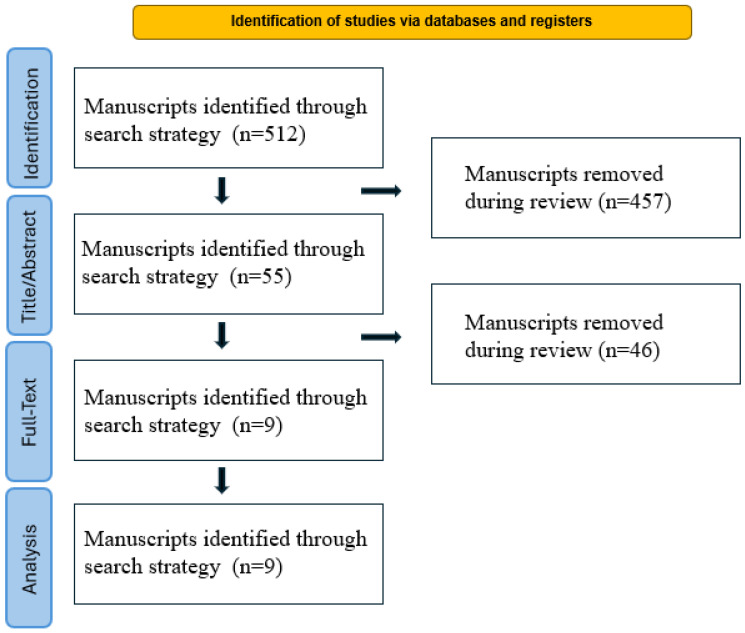
PRISMA flowchart diagram (adapted from Page et al., 2021) [11].

This review was conducted in accordance with the PRISMA 2020 updated guidelines [11], and the study selection process is summarized in the PRISMA flow diagram (Figure 1). A comprehensive literature search was performed on 20th November 2025 using the electronic databases PubMed, Scopus, and Web of Science. All types of peer-reviewed publications, including systematic reviews, meta-analyses, original research articles, and case series, were considered eligible. Single case reports were excluded due to their limited value. The search strategy was based on combinations of Medical Subject Heading (MeSH) terms and free-text keywords as follows: “reflectance confocal microscopy” (or its acronym “RCM”) AND “periocular area” OR “eyelid” AND “tumor”; “optical coherence tomography” (or its acronym “OCT”) AND “periocular area” OR “eyelid” AND “tumor”; “line-field confocal optical coherence tomography” (or its acronym “LC-OCT”) AND “periocular area” OR “eyelid” AND “tumor”. The research was limited to the last 10 years (2015–2025) in order to reflect current clinical practice and technological advancements in non-invasive imaging of this area for skin tumors. Among non-invasive techniques, RCM, OCT and LC-OCT were included as they are the most common employed in clinical practice.

### 2.2. Study Extraction and Synthesis

After duplicate removal, three independent investigators (SC, GG and RS) screened title and abstract to assess the eligibility of the article. The reference list was manually screened to identify additional relevant studies not retrieved by the initial search. Any discrepancies in study selection were resolved through discussion and consensus with a fourth reviewer (CC). Full-text articles were evaluated according to predefined inclusion criteria: studies assessing the use of RCM, OCT, or LC-OCT in the evaluation of skin tumors located in the periocular area or eyelids; availability of sufficient methodological and clinical data. Articles not written in English and single case reports were excluded.

For each included study, the following data were extracted: author(s), year, type of article, study design, imaging technique used, anatomical site, and number of patients included. Extracted data are summarized in Table 1, Table 2 and Table 3. Data extraction was performed by one reviewer (SC) and subsequently verified by the other authors to ensure accuracy and consistency.

Given the heterogeneity of study designs, imaging protocols, outcome measures, and the limited number of available studies, a quantitative meta-analysis was not considered methodologically appropriate. Therefore, a narrative synthesis was conducted, organizing and integrating the results according to each imaging modality (RCM, OCT, and LC-OCT) and their role in the evaluation of periocular skin tumors.

### 2.3. Methodological Considerations

To assess the quality and risk of bias of studies, Quality Assessment of Diagnostic Accuracy Studies-2 (QUADAS-2) was used. This tool comprises 4 domains: patient selection, index test, reference standard, and flow and timing. The risk of bias was scored as unclear, high, or low. Case series were excluded from quality evaluation.

## 3. Results

The literature search retrieved 512 records across the selected databases. After removal of duplicates and exclusion of articles not meeting the predefined criteria, a total of nine studies were included in the final qualitative synthesis. The study selection process is summarized in the PRISMA flow diagram (Figure 1). Studies included were of high quality, assessed using the QUADAS-2 tool.

### 3.1. Reflectance Confocal Microscopy

Four studies evaluated the application of reflectance confocal microscopy (RCM) in the assessment of periocular and eyelid lesions. RCM was mainly investigated in the differential diagnosis of pigmented and non-pigmented tumors, particularly in clinically equivocal cases.

**Figure 2:**
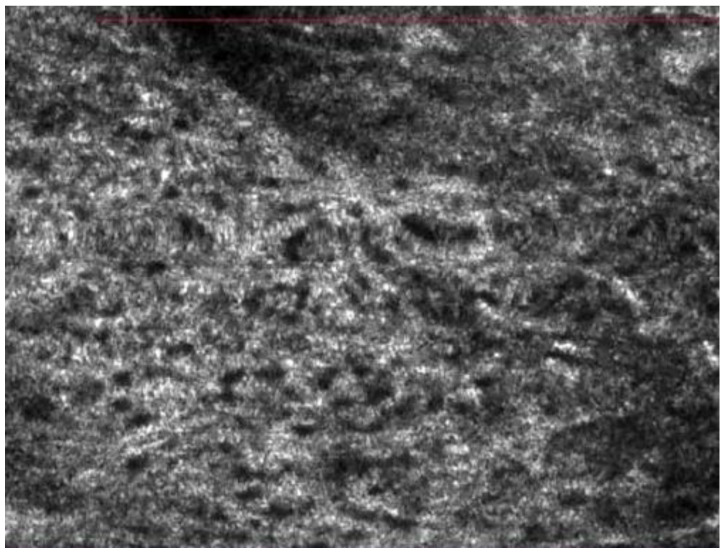
Confocal stack of a squamous cell carcinoma characterized by architectural disarrangement of the epidermis.

Its application provided additional microscopic information beyond clinical and dermoscopic evaluation. Among benign pigmented conditions, features of nevus of Ota were assessed by Grechenig et al., who analyzed 15 patients and described several recurring confocal features that can increase confidence for its diagnosis in doubtful cases [14]. The presence of highly reflective, large and homomorphic dendritic cells was observed among the collagen fibers. These cells displayed either spindle-shaped morphology with two dendrites or multidendritic configurations, often arranged in clusters within a diffuse proliferation. In contrast to common melanocytic nevi, melanocytes in nevus of Ota frequently showed well-defined hyporeflective nuclei. Pagetoid melanocytes, commonly observed in LM and LMM, were consistently absent [14]. Among malignant neoplasms, basal cell carcinoma (BCC) was the most extensively studied tumor using RCM. Key features include the presence of bright tumor islands or dark silhouettes, nuclear streaming, cleft-like dark spaces surrounding tumor nests, and convoluted and dilated blood vessels [12]. Although the diagnostic function of confocal microscopy is now well established in dermatology, it is less so in ophthalmology, a field that frequently first identifies lesions in this area. For this reason, Rubegni et al. assessed the impact of a brief educational intervention to ophthalmologists [13]. After a single day of dermoscopy and RCM training, participants demonstrated a 9% improvement in sensitivity (from 33.3% to 41.7%), while accuracy remained stable (~64%). Notably, management decisions changed substantially: recommendations for dermoscopic follow-up of benign lesions decreased from 51.6% to 22.2%, whereas referrals for RCM increased. In malignant lesions, recommendation for simple follow-up dropped sharply (from 37.0% to 9.9%), with a corresponding increase in biopsy (+12%) and RCM referral (+15%) [13]. In another work, the location of the lesion emerged as a relevant factor influencing diagnostic performance [15]. Indeed, in a large prospective study, Cinotti et al. reported that handheld RCM outperformed the sensitivity of slit lamp examination for detecting malignancies of the eyelid margin (98% vs. 92%) and conjunctiva (100% vs. 88%) (Figure 2). Specificity, however, varied: it was superior for eyelid margin tumors with RCM (74% vs. 46%) but slightly lower for conjunctival malignancies (78% vs. 88%), primarily due to the misinterpretation of hyperreflective Langerhans cells as melanocytes [15].

### 3.2. Optical Coherence Tomography

**Figure 3:**
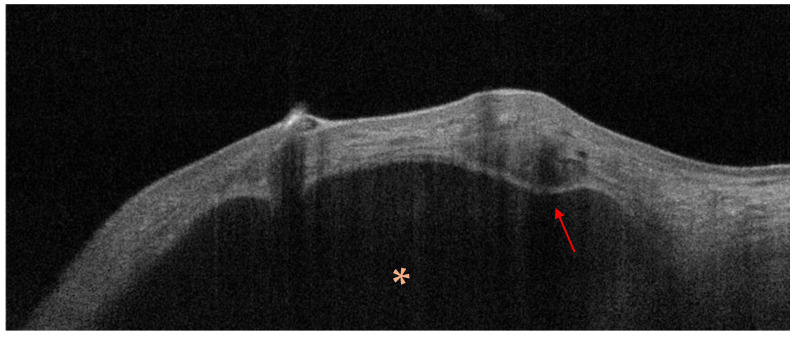
OCT image of a basal cell carcinoma of the lower eyelid showing a round dark structure in the upper dermis (orange asterisk) surrounded by hyperreflective stroma (red arrow).

Optical coherence tomography (OCT) was employed in three studies mainly assessing non-melanoma skin cancers. Bergeron et al. analyzed OCT features of periocular BCC in 38 patients. Loss of dermal epidermal junction was observed in 100% of the cases (38 patients), while hyporeflective tumor nests were observed in 82% of cases (31 patients) [17]. Six additional features not predominant for the diagnosis of BCC were explored and included an accentuated hyperreflective band, ulceration, thickening of the epidermis, hyperreflective nests in the epidermis, bunch of grapes nodules, and cystic structures. In the same study, OCT was also applied to SCC and SGC [17]. In SCC, acanthosis and DEJ loss were observed in 100% of cases, while a hyperreflective band and hyporeflective tumor nests were present in 50%. In sebaceous carcinoma, all lesions showed DEJ loss (5/5), epidermal thickening in 60% of cases, and hyperreflective nests in 60%. In a subsequent publication by Bergeron et al., OCT findings were analyzed in 46 periocular BCCs [16]. Loss of dermo-epidermal junction was again observed in all cases, while hyporeflective tumor nests were identified in 83% (38/46) (Figure 3). Less frequent features included the presence of a hyperreflective band, acanthosis, cystic areas, ulceration and hyperreflective nest. Differently from the previous report, the authors suggested abandoning the “bunch of grapes” feature as it lacked a specific histopathological correlate beyond the presence of small hyporeflective nodules. Both cases of SCC analyzed in this series showed loss of dermo-epidermal junction and acanthosis; however, these findings were acknowledged as non-specific criteria as they can also be observed in other benign and malignant lesions such as actinic keratosis, keratoacanthoma and SGC as well. Similarly, OCT findings for SGC were heterogeneous, displaying loss of dermo-epidermal junction, acanthosis and hyperreflective nests [16].

Pelosini et al. evaluated 15 cases of periocular nodular BCC using in vivo OCT and categorized the main criteria into superficial, intralesional and perilesional features. Superficial changes included epidermal thinning, which was identified in 100% of patients (15/15); crusting and ulceration (5/15, 33%); and decreased density of hair follicles over the BCC nodule (8/15, 53%). Intralesional features consisted of hyporeflective nodules (15/15, 100%), hyperreflective edges (15/15, 100%) and central necrosis (3/15, 20%). Perilesional features were hyporeflective borders (11/15, 73%), hyperreflective margins (15/15, 100%) and dilated blood vessels (5/15, 33%) [18].

### 3.3. Line-Field Confocal Optical Coherence Tomography

The application of LC-OCT in the periocular skin area was assessed in two studies [8,10].

**Figure 4:**
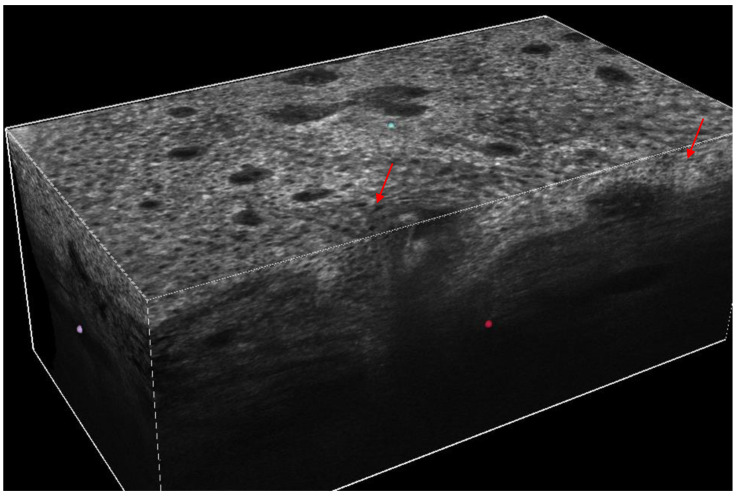
3D LC-OCT cube of a melanoma in situ on the lower eyelid exhibiting roundish atypical cells with a prevalent perifollicular distribution (red arrows).

Di Stefani et al. described the LC-OCT features of various benign and malignant periocular skin tumors, also comparing in vivo imaging findings with histopathology [8]. LC-OCT enabled the identification of lesion-specific morphological characteristics, showing a high concordance with histological examination [8]. BCCs were recognized for the observation of dermal lobules corresponding to tumor islands and consisting of a grey area defined as a hyper-/hyporeflective structure with different shapes, featuring the so-called “millefeuille pattern”. Additional features including peritumoral vessels and epidermal changes have been observed according to BCC subtype [8]. SCC was identified for the presence of architectural alterations as disarranged epidermis with atypical keratinocytes, hyperkeratosis, and acanthosis impeding the visualization of DEJ in invasive SCC. In cutaneous melanoma, LC-OCT were characterized by an irregular honeycomb pattern and large, bright, roundish, and dendritic atypical cells along the basal layer and upward in the epidermis [8] (Figure 4). Comparable results were reported by Verzì et al., who analyzed skin lesions located on the upper or lower eyelid margins [10]. In both studies, LC-OCT allowed in vivo visualization of key microscopic features relevant for diagnosis. The authors pointed out that beyond diagnostic skills, LC-OCT fits a probe perfectly suitable for the examination of lesions in this particular anatomic region, as its dimension may be easily positioned and rapidly moved, also on eyelid margin for real-time evaluation and image acquisition [8,10].

## 4. Discussion

The periocular region represents one of the most challenging anatomical areas for diagnosis and therapeutical approach. Its complex anatomy, functional relevance, and aesthetic importance require a careful balance between diagnostic accuracy and tissue preservation [1,2]. In this context, the present review provides a comprehensive overview of the current evidence evaluating the use of non-invasive imaging techniques. Overall, the available literature consistently indicates that non-invasive imaging may represent a valuable adjunct to clinical examination and dermoscopy to refine pre-treatment assessment, guide clinical decision-making, and potentially reduce unnecessary biopsies when lesions present with specific features.

RCM emerged as the most extensively investigated modality in the periocular setting. Its near-cellular resolution allows for detailed evaluation of epidermal and superficial dermal structures, making it particularly useful in the differential diagnosis of pigmented lesions and basal cell carcinoma. The studies highlighted the ability of RCM to identify diagnostic features of periocular BCC and to distinguish malignant melanocytic lesions from benign simulators, including conditions characterized by dermal hypermelanocytosis. Additionally, evidence suggests that lesion location may influence diagnostic performance, with higher sensitivity observed for eyelid margin tumors compared with conjunctival lesions. This gap is supposed to be due to an easier examination of eyelid margins. These findings underline the importance of anatomical context when interpreting confocal images and reinforce the need for specific training in periocular RCM interpretation.

Beyond diagnostic accuracy, RCM also demonstrated an impact on clinical management. Educational studies showed that even limited training may modify diagnostic confidence and management strategies, reducing reliance on follow-up alone and increasing appropriate referrals for advanced imaging or biopsy. This aspect is particularly relevant in interdisciplinary settings, where ophthalmologists are often the first clinicians to evaluate periocular lesions [12,13,14,15].

OCT was primarily investigated in the assessment of non-melanoma skin cancers, especially BCC. Across multiple studies, consistent OCT features included loss of the dermo-epidermal junction and the presence of hyporeflective tumor nests. However, the specificity of several OCT findings remains limited, as similar features may be observed in both malignant and benign conditions. The heterogeneity of reported OCT patterns in squamous cell carcinoma and sebaceous carcinoma suggests that OCT should be interpreted cautiously and as complementary to clinical features and dermoscopy. Nevertheless, OCT offers the advantage of greater imaging depth compared with RCM, allowing for visualization of deeper tumor components, which may be relevant for pre-surgical planning [16,17,18].

LC-OCT represents the most recent advancement and is currently supported by limited although promising scientific evidence. By combining vertical and horizontal imaging with high spatial resolution, LC-OCT appears capable of bridging some of the technical weaknesses currently existing in RCM and conventional OCT. Indeed, the potential afforded by the new LC-OCT to scan, in real time, both vertical and horizontal plane, as well as to build 3D cubes, seems ideal for the investigation of this anatomical region, as this device can perform a whole examination of the lesion up to the superficial/mid-dermis. Studies investigating LC-OCT in this area suggested its valuable role in non-invasive diagnosis due to high similarity to histopathology, providing a real-time “virtual biopsy”. Moreover, the compact probe design and ease of positioning along the eyelid margin are practical advantages in clinical routine [8,10].

Several limitations of the existing literature must be mentioned. First, the overall number of studies specifically addressing periocular lesions remains limited, with considerable heterogeneity in study design, sample size, lesion types, and imaging protocols. Considering the OCT features, there is an overlap between benign and malignant lesions, which limits the specificity; additionally, OCT description of squamous cell carcinoma and sebaceous carcinoma are based on very small sample sizes. As with regard to the LC-OCT technique, evidence is limited to two investigations.

Second, most studies were observational and descriptive in nature, precluding quantitative synthesis or robust comparative analysis. Third, operator dependency and the need for specialized training represent relevant barriers to widespread implementation, particularly for high-resolution techniques such as RCM and LC-OCT. These limitations justify the choice of a narrative synthesis and highlight the need for larger, prospective studies with standardized imaging criteria. It should be also mentioned that data extraction was primarily performed by a single reviewer, which may increase the risk of extraction bias.

When integrated into a multimodal assessment strategy, these tools may enhance diagnostic confidence, support interdisciplinary collaboration, and assist in therapeutic planning, especially in cases where tissue preservation is paramount.

Future research should focus on prospective, multicenter studies aimed at standardizing imaging criteria, defining diagnostic thresholds, and clarifying the impact of non-invasive imaging on clinical outcomes.

## 5. Conclusions

Non-invasive imaging techniques represent a valuable adjunct in the evaluation of periocular skin tumors, an anatomical region where diagnostic accuracy and tissue preservation are of paramount importance. The available evidence indicates that non-invasive imaging can enhance lesion characterization when clinical and dermoscopic findings are inconclusive. The appropriate integration into a multimodal workflow strategy may support interdisciplinary collaboration and assist in therapeutic planning. However, the current evidence is preliminary and is largely based on small observational studies with limited head-to-head comparisons.

## 6. Future Directions

These techniques should be regarded as complementary tools rather than replacements for histopathological examination. Their integration into a multimodal diagnostic approach may improve clinical confidence, support interdisciplinary management, and potentially reduce unnecessary invasive procedures in appropriately selected patients. Further prospective studies with standardized protocols along with training courses are warranted to better define their diagnostic performance and clinical impact in periocular oncology.

## Figures and Tables

**Table 1:** Reflectance confocal microscopy.

Authors	Title	Year	Type of Article	Design of the Study	Number of Patients
Caviglia M et al. [12]	A Systematic Review and Meta-Analysis of Ocular and Periocular Basal Cell Carcinoma with First-Time Description of Dermoscopic and Reflectance Confocal Microscopy Features of Caruncle Basal Cell Carcinoma	2025	Original study	Systematic review and meta-analysis	71,730
Rubegni G et al. [13]	Dermoscopy Training Course Improves Ophthalmologists’ Accuracy in Diagnosing Atypical Pigmented Periorbital Skin Lesions	2024	Original study	Retrospective, multicentric	80
Grechenig C et al. [14]	Examination of the melanocytes of the Nevus of Ota with in vivo reflectance confocal microscopy: 15 cases	2018	Case series	Prospective, multicentric	15
Cinotti E et al. [15]	Handheld In Vivo Reflectance Confocal Microscopy for the Diagnosis of Eyelid Margin and Conjunctival Tumors	2017	Original study	Prospective, monocentric	278

**Table 2:** Optical coherence tomography.

Authors	Title	Year	Type of Article	Design of the Study	Number of Patients
Di Stefani A et al. [8]	Line-Field Confocal Optical Coherence Tomography Evaluation of Eyelid Skin Lesions.	2023	Original study	Retrospective, monocentric	51
Verzì AE et al. [10]	Line-field confocal optical coherence tomography of eyelid margin growths: A case series.	2024	Case series	Retrospective, monocentric	28

**Table 3:** Line-field confocal optical coherence tomography.

Authors	Title	Year	Type of Article	Design of the Study	Number of Patients
Bergeron S. et al. [16]	Optical Coherence Tomography of Peri-Ocular Skin Cancers: An Optical Biopsy	2021	Original study	Prospective, monocentric	57
Bergeron S. et al. [17]	Novel application of anterior segment optical coherence tomography for periocular imaging	2019	Original study	Prospective, multicentric	50
Pelosini L. et al. [18]	A novel imaging approach to periocular basal cell carcinoma: in vivo optical coherence tomography and histological correlates	2015	Original study	Prospective, monocentric	15

## Data Availability

Data are available on request from the authors.
